# Genetic diversity, population structure, and DNA fingerprinting of *Ailanthus altissima* var. *erythrocarpa* based on EST-SSR markers

**DOI:** 10.1038/s41598-023-46798-2

**Published:** 2023-11-07

**Authors:** Manman Zhang, Conghui Zheng, Jida Li, Xueyong Wang, Chunpeng Liu, Xiangjun Li, Zhenhua Xu, Kejiu Du

**Affiliations:** 1 Hebei Agricultural University, Baoding, 071000 Hebei China; 2 Hebei Technical Innovation Center for Forest Improved Variety, Shijiazhuang, 050061 Hebei China; 3 Hebei Academy of Forestry and Grassland Sciences, Shijiazhuang, 050061 Hebei China

**Keywords:** Genetics, Molecular biology

## Abstract

*Ailanthus altissima* var. *erythrocarpa* is an *A. altissima* variety with high economic, ecological and ornamental value, but there have been no reports on the development of SSR primers for it. According to the SSR primer information provided by the transcriptome of *A. altissima* var. *erythrocarpa*, 120 individuals with different redness levels were used to screen polymorphic primers. Transcriptomic analysis revealed 10,681 SSR loci, of which mononucleotide repeats were dominant (58.3%), followed by dinucleotide and trinucleotide repeats (16.6%, 15.1%) and pentanucleotide repeats (0.2%). Among 140 pairs of randomly selected primers, nineteen pairs of core primers with high polymorphism were obtained. The average number of alleles (*Na*), average number of effective alleles (*Ne*), average Shannon’s diversity index (*I*), average observed heterozygosity (*Ho*), average expected heterozygosity (*He*), fixation index (*F*) and polymorphic information content (*PIC*) were 11.623, 4.098, 1.626, 0.516, 0.696, 0.232 and 0.671, respectively. Nineteen EST-SSR markers were used to study the genetic diversity and population structure of *A. altissima* var. *erythrocarpa*. The phylogenetic tree, PCoA, and structure analysis all divided the tested resources into two categories, clearly showing the genetic variation between individuals. The population showed high genetic diversity, mainly derived from intraspecific variation. Among nineteen pairs of primers, 4 pairs (p33, p15, p46, p92) could effectively distinguish and be used for fingerprinting of the tested materials. This study is of great significance for genetic diversity analysis and molecular-assisted breeding of *A. altissima* var. *erythrocarpa.*

## Introduction

*Ailanthus altissima* (Mill) Swingle, belonging to Simaroubaceae and *Ailanthus*, is widely distributed on all continents except Antarctica and is a pioneer tree used in greening and barren mountain afforestation. It has high economic, medicinal, ecological and ornamental value^1–7^. Wild related species often become important resources for innovative plant breeding due to their higher resistance and richer genetic characteristics than those of this species. *Ailanthus altissima* var. *erythrocarpa* is a variety of *A. altissima* (Mill) Swingle; it is usually recognized as a single population or multiple very small populations in the northern region of China. *A. altissima* var. *erythrocarpa* has significantly better physical and chemical properties^8^ and medicinal and ornamental value than *A. altissima* (Mill) Swingle due to its higher contents of flavonoids and other substances. The fruiting period of *A. altissima* var. *erythrocarpa* is from June to September. Because the fruit is red and persists for months, it has high ornamental value and a high profile^8^. There are significant differences in the appearance time, duration and chroma of fruit color (data not yet published). Genetic composition and the environment can lead to stability or variability in plant morphology^9^, which necessitates scientific identification and evaluation of germplasm of *A. altissima* var. *erythrocarpa.*

Expressed Sequence Tag-SSRs (EST-SSRs) are derived from mRNA transcription sequences and are applicable to genetic linkage map construction, genetic diversity analysis, population genetic structure research, and studies of species evolution and variation in plants with or without reference genomes^10–12^. Compared with g-SSRs, EST-SSRs are more efficient, require less time and have a lower cost. They also have stronger transferability among species^13–19^ and have been widely used in plant variety identification, genetic structure analysis and fingerprint development^20–24^. To date, due to the lack of genomic and genetic information, there have been few studies on the genetic diversity of *A. altissima* (Mill) Swingle in China and elsewhere. The research that has been performed concerned the development of nuclear microsatellite markers for *Ailanthus* on Mediterranean islands and in eastern Austria; population structure and genetic diversity analyses of North American *A. altissima* (Mill) Swingle; patterns of regional expansion and genetic differentiation among populations of Japanese highland *A. altissima* (Mill) Swingle; the origin, expansion and genetic structure of Chinese squash introduced from Austria; the development of 10 cpSSR markers and 13 EST-SSR markers; and genetic diversity analysis of 14 population groups in different regions of China^18,25–32^. These findings have promoted the application of SSR labeling in *A. altissima* (Mill) Swingle. Nevertheless, there are few reports on the genetic diversity of *A. altissima* var. *erythrocarpa* and no reports on the development of polymorphic SSR primers or fingerprints.

The number of available expressed sequence tags in *A. altissima* (Mill) Swingle is limited, and EST-SSRs of *A. altissima* var. *erythrocarpa* have not been reported. Therefore, transcriptome sequencing was used to provide SSR marker information for *A. altissima* var. *erythrocarpa*. We developed a total of nineteen pairs of EST-SSR markers and conducted genetic diversity and population structure analyses on the basis of 120 wild individuals of *A. altissima* var. *erythrocarpa* from 7 regions in China, constructing fingerprint maps. This study will provide technical support for resource collection and protection, germplasm bank establishment and management, germplasm identification and intellectual property protection in the future and will lay a theoretical foundation for breeding and germplasm innovation of *A. altissima* var. *erythrocarpa* and *A. altissima* (Mill) Swingle.

## Results

### Number and distribution of EST-SSR loci in *A. altissima* var. *erythrocarpa*

**Figure 1:**
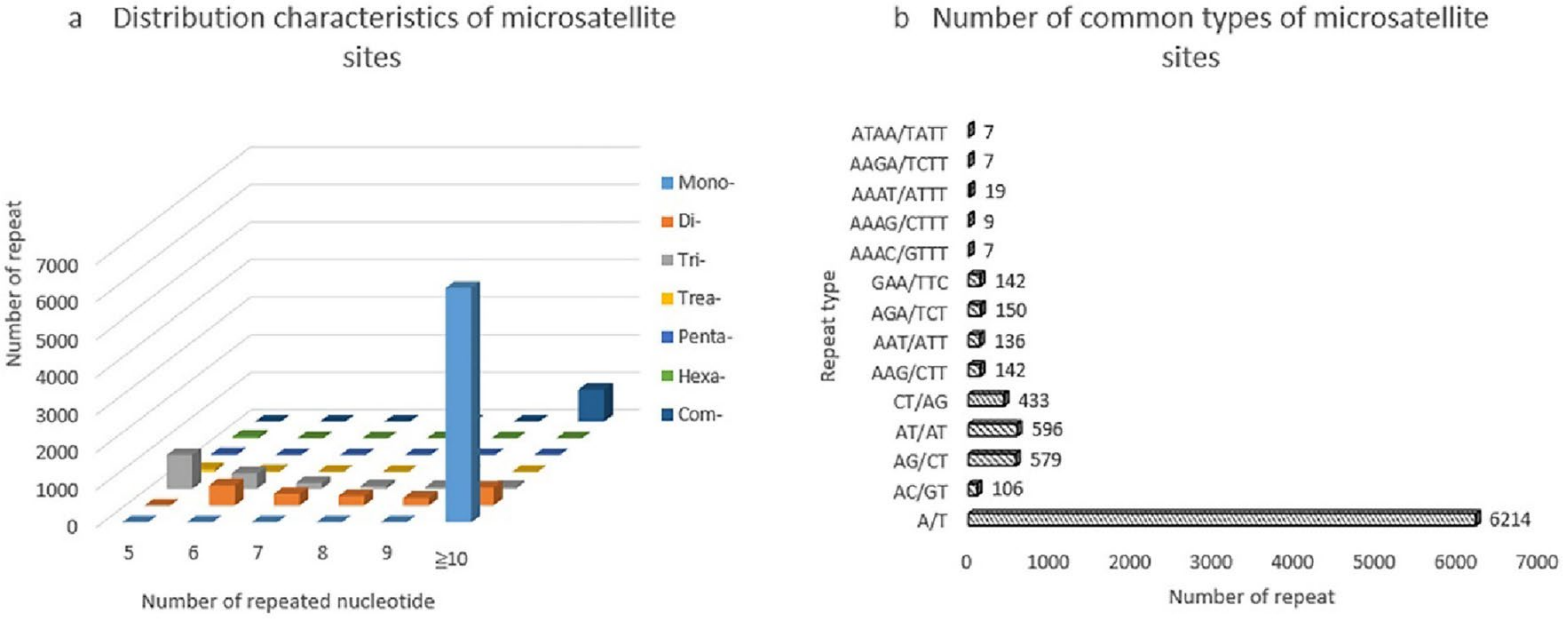
Distribution of different types of EST-SSRs in *A. altissima* var. *erythrocarpa*. *Mono* mononucleotide repeat motif, *Di* dinucleotide repeat motif, *Tri* Trinucleotide repeat motif, *Tetra* tetranucleotide repeat motif, *Penta* pentanucleotide repeat motif, *Hexa* hexanucleotide repeat motif, *Com* compound nucleotide repeat motif.

By using the Illumina HiSeq™ high-throughput sequencing platform, transcriptome sequencing of *A. altissima* var. *erythrocarpa* was performed, and an SSR search of the spliced and assembled unigenes revealed 9828 accurate SSRs and 853 compound SSRs, totaling 10,681 SSR loci. The EST-SSR types of *A. altissima* var. *erythrocarpa* were abundant, and their distribution showed (Fig. 1a) trinucleotide repeats, dinucleotide repeats and mononucleotide repeats in the largest numbers, among which 6228 mononucleotides accounted for the highest proportion (58.31%); 1777 dinucleotides accounted for 16.64%; 1621 trinucleotides accounted for 15.18%; and tetra-hexanucleotide repeats were rare, accounting for 0.99%, 0.24% and 0.66%, respectively. Other complex nucleotide repeats accounted for 7.99% of the total SSRs, indicating that mono-trinucleotides were the main repeat motifs of EST-SSRs in *A. altissima* var. *erythrocarpa*.

According to EST-SSR repeat motif type analysis, there were 166 exact SSR motifs, and the numbers of mononucleotide to hexanucleotide repeats were 2, 6, 30, 39, 23 and 66, respectively (Table 1). Mononucleotides were mainly the A/T repeat type (6214), accounting for 58.18% of the total number of mononucleotides. Dinucleotides were mainly the AT/AT (33.54%) and AG/CT (32.58%) repeat motifs, accounting for 5.58% and 5.42% of the total SSRs, respectively. The specific percentages of the most common trinucleotides, AGA/TCT, AAG/CTT and CTT/AAG, were 1.40%, 1.35% and 1.33%, respectively. The AAAT/ATTT and AAAG/CTTT tetranucleotides accounted for 0.18% and 0.08%, respectively. The penta- to hexanucleotide repeat motifs appeared in many forms, but their frequency was relatively low. Among all repeat types (Fig. 1b), the number of repeats of SSR loci motifs was 5 or more, and the frequency of nucleotide repeats decreased gradually with an increase in the number of repeats. The types with more than 10 repetitions accounted for 63.16% of the total, the largest percentage. This was followed by those with 5 and 6, accounting for 9.90% and 9.14% of the total, respectively. The lowest percentage was observed for those with 9 repetitions, accounting for only 2.32%. The number of mononucleotide repeats was 10 or more, that of dinucleotides was 6 or more, and the highest repetition rate was 6 times. The trinucleotide frequency was highest for 5 or more repetitions. The number of tetra- to hexanucleotides was mainly 5, which indicated that the polymorphism potential of *A. altissima* var. *erythrocarpa* was high.

**Table 1:** Types of SSR motifs in *A. altissima* var. *erythrocarpa.*

Repeat motif length	Repeat motif and percentage (%)	Total
Mononucleotide	A/T + C/G (58.31)	2
Dinucleotide	AC/GT (0.99), AG/CT (5.42), AT/AT (5.58), CT/AG (4.05), other (0.59)	6
Trinucleotide	AAG/CTT (1.35), AAT/ATT (1.27), AGA/TCT (1.40), CTT/AAG (1.33), other (9.83)	30
Tetranucleotide	AAAC/GTTT (0.07), AAAG/CTTT (0.08), AAAT/ATTT (0.18), AAGA/TCTT (0.07), ATAA/TTAT (0.07), other (0.52)	39
Pentanucleotide	AAATA + AAATC + AAGCC + ATCAA + ATGAG + ATTGG + CACTA + CCAAA + CCAGT + CCCCA + CGAAA + CTGAT + CTTTT/AAAAG + GAAAT + GAGAA + GTTGT + TAAAA + TCTCC + TCTTT + TGAGT + TGATG + TTTCT + TTTGA (0.24)	23
Hexanucleotide	AAAAAG + AAAACA + AAAATA + AAGAAA + AAGAAC + AATCAA + ACCAAC + AGAAAA + AGAAAG + AGCAAG + AGCAGG + AGCCTG + ATAGTC + ATATTG + ATGAAT + ATGTAA + ATGTTT + ATTCGC + ATTTAC + ATTTTG + CAAACC + CAAATC + CAACAC + CAACAG + CAAGAA + CAGAGA + CATCTT + CATGGA + CCCAAT + CCTGAG + CCTTAA + CGCCAT + CTGAAC + CTTCAT + GAGAAG + GAGCAG + GGAGAA + GGCTCA + GGTCTC + GTGGGA + GTGGGC + GTTCTG + GTTGAA + GTTTTC + TAATAC + TATTGA + TCAGAT + TCATCC + TCCTCT + TCGGTA + TCTGGC + TGAAAA + TGACTG + TGCTGG + TGGGAC + TGGTGC + TGTGCG + TGTTCT + TGTTTT + TTCTGA + TTATGC + TTGGTT + TTGTTC + TTTGCC + TTTGTG + TTTTGG (0.66)	66
Compound Nucleotide	7.99	

### Screening and validation of EST-SSR primers for *A. altissima* var. *erythrocarpa*

Based on transcriptome sequencing data analysis, SSR primers were designed, their information was obtained, and 140 pairs of primers were randomly selected for effectiveness verification. Initial screening was performed by PCR amplification and agarose gel electrophoresis, in which 106 pairs of primers were able to amplify bands with the expected fragment size, eventually resulting in amplification in 120 *A. altissima* var. *erythrocarpa* materials. DNA samples were tested by polyacrylamide gel electrophoresis and capillary electrophoresis. The analysis results for nineteen polymorphic primers (Table 2) showed clear and rich polymorphisms. The effective amplification rate was 75.71%, and the primer polymorphism rate was 13.57%.

**Table 2:** Information on polymorphic SSR primers.

Primer No	Repeat motif	Primer sequence (5′–3′)	Expected size (bp)	Tm (°C)
p15	(TA)9	AGGGAAGCTCCGAGTAAGGA	280	59
		CCTTCCCTTGGGAATCTGCA		
p23	(ATA)5	TGGCGGAATAATGCTCCACT	250	59
		GCCTTCTGGGATTTCCGACA		
p30	(TTC)7	TCAACAAATTGCGCCAAGGG	267	59
		GGGTTTCTGTGGTGTTTGGG		
p31	(CTTT)5	TGGTTACTTGTAAACCCAGCCA	158	60
		ACTCACCGACCCAGAAACAA		
p33	(TTCT)5	CGCCAGCGTTCTCTTTCATC	220	59
		GCAGATTTCCGCCATCGAGA		
p40	(TTAT)5	GAAGGGTGACATGCCAGTCA	227	60
		GCGTGGGGTGATAGTAAGCA		
p46	(CACTA)5	TGCAATTGCGATGCCAACAA	231	59
		ATCCCAAAAGCTGCGACAGT		
p49	(AAGCC)5	ATGTGAGTGCCAAGACGGAG	254	60
		ACGGAATTCGAAGCACCTGT		
p53	(GGAGAA)7	AGGAGAGAACGGGACCATGA	239	59
		CGATCTCGCAACTCCCTCTC		
p54	(CAAACC)5	ATTCCCTTTACCGCAGAGGC	274	60
		GGTGGATGAAGGGCTGACTC		
p56	(AGAAAG)5	GAACCAAAGGCCCCTTCAGT	194	60
		ATTCGGAATTTCGGGGCAGT		
p69	(T)11*(A)12	CACTGACACGCTCACCTTCT	181	60
		GGCAGATACCGACGTTAGGG		
p76	(AT)7(TATC)6*	AGAAAAGGGCTGAGAGTGGC	264	57
		CCATTTATTAAACATCCATAAGAGGGA		
p79	(A)10(AC)6*(A)10	TAAACCCCTACCGTGCGTTC	234	60
		GAACTCTGCCAACAACGCTG		
p92	(CCG)9	CACCATGTCCACCGCCTTAT	267	60
		TTGCGCTCTGATGATCCACA		
p95	(TGT)6	CTCCCCACGATTTGCCTGAT	274	60
		ATTTGTCCCTCGACGACTCG		
p111	(GAAAT)5	CAGTTCTTCCAAGTGCACAAA	238	58
		TGGTAGGTGGCATCCATTACT		
p125	(TG)9(TA)8	ATCAGACCAATGTGCAGGGG	185	60
		CTCTCTCGGTGTGCATGTGT		
p127	(T)13*(TC)6	ACAGAGTGCCCTTATCGTGT	218	58
		ATCCAATCGTTTCCGGCCAT		

∗ Compound SSR loci.

### Primer polymorphism analysis

**Figure 2:**
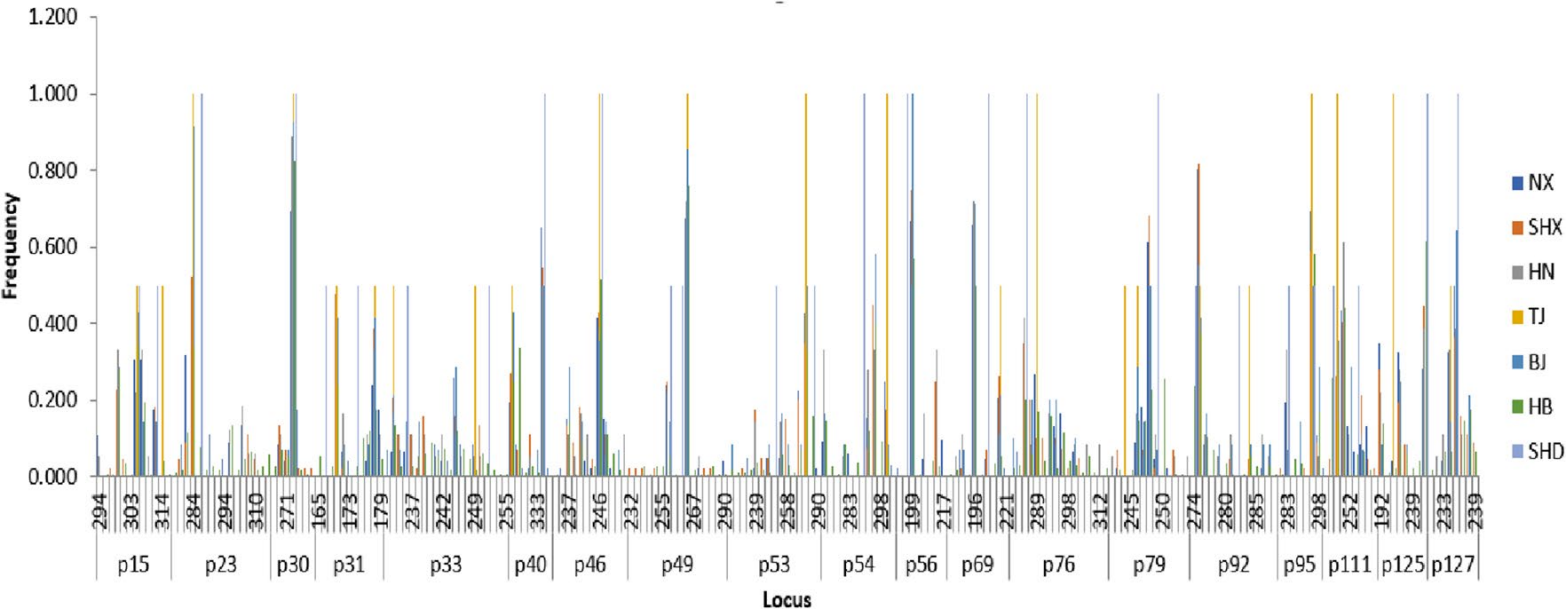
Allelic distribution patterns of nineteen polymorphic loci in 7 natural populations.

Based on capillary electrophoresis, correlation analysis of 7 natural populations of *A. altissima* var. *erythrocarpa* was carried out with microsatellite sites showing polymorphism (Table 3 and Fig. 2). There were 221 alleles identified among the nineteen EST-SSR markers. All 120 individuals could be uniquely genotyped using these 221 alleles, demonstrating the high discrimination capacity of the nineteen EST-SSR markers. The markers showed considerable variation, with the number of alleles (*Na*) ranging from 7 to 20 (mean 11.632), number of effective alleles (*Ne*) from 1.550 to 11.942 (mean 4.098), observed heterozygosity (*Ho*) from 0.191 to 0.931 (mean 0.516), expected heterozygosity (*He*) from 0.355 to 0.916 (mean 0.696), unbiased expected heterozygosity (uHe) from 0.356 to 0.920 (mean 0.699), Shannon’s information index (*I*) from 0.797 to 2.675 (mean 1.626), and polymorphism information content (*PIC*) from 0.340 to 0.912 (mean 0.671). The marker with the most alleles was p33 (20), and the markers with the fewest alleles were p30, p40 and p95 (7). The *PIC* values of the nineteen EST-SSR markers ranged from 0.340 to 0.912, with an average of 0.671. All markers exhibited high polymorphism, with the exception of p30 and p49 (*PIC* > 0.5).

**Table 3:** Genetic diversity parameters for *A. altissima* var. *erythrocarpa* individuals at the nineteen microsatellite markers.

Locus	*N*	*Na*	*Ne*	*I*	*Ho*	*He*	*uHe*	*F*	*PIC*
p15	120	12	4.520	1.727	0.800	0.779	0.782	− 0.027	0.745
p23	112	16	4.743	2.036	0.259	0.789	0.793	0.672	0.773
p30	120	7	1.550	0.797	0.333	0.355	0.356	0.061	0.340
p31	116	11	4.900	1.889	0.931	0.796	0.799	− 0.170	0.771
p33	118	20	11.942	2.675	0.873	0.916	0.920	0.047	0.912
p40	115	7	2.933	1.258	0.852	0.659	0.662	− 0.293	0.603
p46	120	12	3.624	1.685	0.892	0.724	0.727	− 0.231	0.742
p49	119	16	1.874	1.142	0.857	0.466	0.468	− 0.838	0.443
p53	105	15	5.010	2.035	0.429	0.800	0.804	0.465	0.784
p54	107	12	4.328	1.807	0.570	0.769	0.773	0.259	0.744
p56	115	8	2.220	1.130	0.191	0.550	0.552	0.652	0.510
p69	116	10	2.499	1.238	0.345	0.600	0.602	0.425	0.556
p76	95	16	7.462	2.300	0.253	0.866	0.871	0.708	0.854
p79	119	13	4.195	1.748	0.303	0.762	0.765	0.603	0.736
p92	119	14	2.957	1.645	0.361	0.662	0.665	0.454	0.642
p95	119	7	2.350	1.135	0.630	0.575	0.577	− 0.097	0.529
p111	114	9	4.039	1.670	0.395	0.752	0.756	0.475	0.724
p125	105	8	3.111	1.401	0.295	0.679	0.682	0.565	0.636
p127	117	8	3.609	1.574	0.231	0.723	0.726	0.681	0.693
Mean	114.263	11.632	4.098	1.626	0.516	0.696	0.699	0.232	0.671

As shown in Table 4, the average inbreeding coefficients *Fis* and *Fit* were − 0.018 and 0.199, respectively, indicating that the number of homozygous individuals was greater than that of heterozygous individuals in the seven natural populations of *A. altissima* var. *erythrocarpa*. Among the seven natural populations, the *Fst* differentiation coefficient of the nineteen EST-SSRs showed a variation range of 0.084–0.456, with an average value of 0.237, which is between 0.15 and 0.25. The variation range of gene flow at polymorphic sites was 0.298–2.737, with an average value of 1.056.

**Table 4:** F-statistics of different microsatellite markers in *A. altissima* var. *erythrocarpa.*

Locus	*Fis*	*Fit*	*Fst*	*Nm*
p15	− 0.227	− 0.103	0.136	1.588
p23	0.525	0.699	0.366	0.433
p30	0.026	0.108	0.084	2.737
p31	− 0.465	− 0.233	0.158	1.331
p33	− 0.208	− 0.025	0.152	1.397
p40	− 0.613	− 0.395	0.135	1.604
p46	− 0.562	− 0.329	0.149	1.429
p49	− 1.406	− 0.750	0.273	0.666
p53	0.301	0.463	0.232	0.829
p54	0.152	0.468	0.372	0.421
p56	0.524	0.741	0.456	0.298
p69	− 0.087	0.254	0.314	0.546
p76	0.610	0.719	0.280	0.641
p79	0.290	0.514	0.315	0.542
p92	− 0.039	0.202	0.232	0.828
p95	− 0.255	− 0.111	0.115	0.922
p111	0.210	0.430	0.278	0.648
p125	0.487	0.658	0.333	0.500
p127	0.394	0.472	0.128	1.696
Mean	− 0.018	0.199	0.237	1.056

### Development of DNA fingerprints for *A. altissima* var.* erythrocarpa*

According to the genotype statistics of nineteen pairs of SSR primers, the primers with higher *PIC* values and more genotypes were selected to distinguish 120 *A. altissima* var. *erythrocarpa* accessions. According to the *PIC* values of the SSR primers, the test materials were added successively from high to low and identified gradually until they were completely separated. Table 5 shows that SSR primers p33 and p15 could distinguish 92.5% of the materials (111). SSR primers p33, p15 and p46 could distinguish 99.17% of the materials (119). Four pairs of SSR primers, p33, p15, p46 and p92, could be used to distinguish the 120 test materials. To facilitate promotion and application, the fingerprint information and names of 120 individuals of *A. altissima* var. *erythrocarpa* were imported into QR code software to generate fingerprint QR codes, wherein the primers p33, p15, p46 and p92 were represented by A, B, C and D, respectively (Supplementary Fig. 1). Please see the appendix for all fingerprints. In this study, nineteen pairs of core primers were screened to completely separate 120 test samples of *A. altissima* var. *erythrocarpa*, and the obtained DNA fingerprints were used to evaluate and identify the genotypes of this variety at the molecular level.

**Table 5:** Differentiation of 120 *A. altissima* var. *erythrocarpa* accessions by adding SSR marker primer combinations one by one.

Primer combination	Number of materials distinguished	Differentiation rate
p33 + p15	111	92.5
p33 + p15 + p46	119	99.17
p33 + p15 + p46 + p92	120	100

### Genetic diversity analysis of *A. altissima* var. *erythrocarpa*

**Figure 3:**
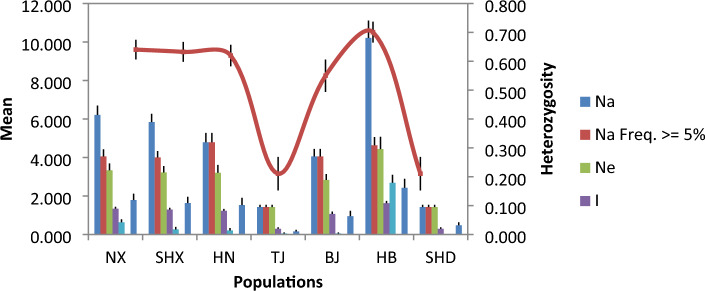
Allelic patterns across populations of *A. altissima* var. *erythrocarpa.*

**Figure 4:**
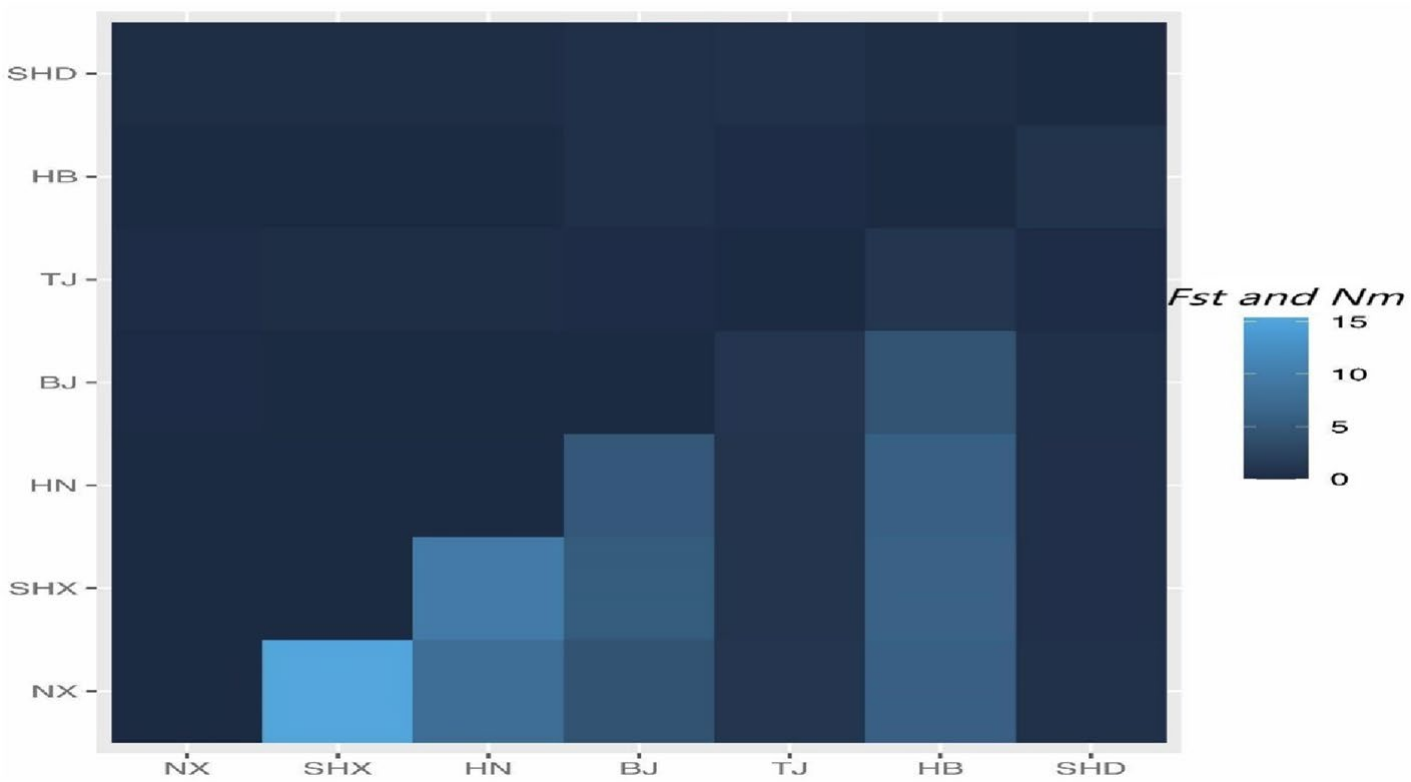
*Fst* and *Nm* values detected for population pairs of *A. altissima* var. *erythrocarpa. Fst*: above diagonal; *Nm*: below diagonal.

There were significant differences in genetic diversity among *A. altissima* var. *erythrocarpa* from 7 different populations (Table 6 and Fig. 3). Compared with those of *A. altissima* var. *erythrocarpa* in the other six populations, the values of *Ne*, *I*, *He*, *uHe* and* F* in the HB population were the highest, and the values of Ho were the lowest. There were differences in indicators of genetic diversity between populations: HB (*Ne* = 4.439) > NX (*Ne* = 3.330) > SHX (*Ne* = 3.224) > HN (*Ne* = 3.202) > BJ (*Ne* = 2.829) > TJ and SHD (*Ne* = 1.421). In contrast, the level of genetic diversity in SHD and TJ was low, which may be due to the small number of individuals. The fixation index was less than 0 in populations TJ, BJ and SHD, indicating that the number of heterozygotes was greater than that of homozygotes, while the number of homozygotes was greater than that of heterozygotes in the other four populations. The average paired *F*_*ST*_ coefficient among the 7 populations of *A. altissima* var. *erythrocarpa* was 0.163. The differentiation between TJ and SHD was the highest (0.600), which may be related to too few samples. The differentiation between BJ and SHD was the second highest (0.342), and the differentiation coefficient between NX and SHX (0.016) was the lowest, but their *Nm* was the highest (15.401), indicating that the genetic differentiation between the NX and SHX populations was small and that gene exchange is frequent (Fig. 4 and Supplementary Table 2).

**Table 6:** Genetic diversity parameters in seven natural populations of *A. altissima* var.* erythrocarpa.*

Population	Sample size	*N*	*Ne*	*I*	*Ho*	*He*	*uHe*	*F*
NX	23	21.789	3.330	1.339	0.556	0.640	0.656	0.111
SHX	22	21.263	3.224	1.294	0.547	0.632	0.648	0.121
HN	9	8.684	3.202	1.225	0.573	0.620	0.658	0.058
TJ	1	1.000	1.421	0.292	0.526	0.211	0.421	− 1.000
BJ	7	6.526	2.829	1.066	0.557	0.550	0.596	− 0.119
HB	57	54.000	4.439	1.625	0.473	0.701	0.707	0.290
SHD	1	1.000	1.421	0.292	0.421	0.211	0.421	− 1.000
Mean (± SE)	–	16.323 (± 1.513)	2.838 (± 0.161)	1.019 (± 0.056)	0.522 (± 0.032)	0.509 (± 0.024)	0.587 (± 0.028)	− 0.065 (± 0.054)

### Population Structure of *A. altissima* var. *erythrocarpa*

The range of genetic distances between the seven natural populations of *A. altissima* var. *erythrocarpa* was 0.042–1.386, and the variation range of genetic similarity was 0.250–0.938 (Table 7). Among the populations, NX and SHX had the highest genetic similarity, and the genetic distance was relatively short, indicating that they were more genetically similar than other pairs. TJ and SHD had the highest genetic similarity and the greatest genetic distance, making them the most distant relatives.

**Table 7:** Nei’s genetic identity (*GI*, above diagonal) and genetic distance (*GD*, below diagonal) between populations.

Pop	NX	SHX	HN	TJ	BJ	HB	SHD
NX		0.938	0.893	0.669	0.850	0.840	0.375
SHX	0.064		0.917	0.660	0.887	0.852	0.363
HN	0.114	0.086		0.654	0.870	0.844	0.363
TJ	0.042	0.415	0.425		0.702	0.641	0.250
BJ	0.163	0.120	0.139	0.354		0.853	0.359
HB	0.175	0.160	0.170	0.445	0.159		0.564
SHD	0.982	1.012	1.014	1.386	1.024	0.573	

**Figure 5:**
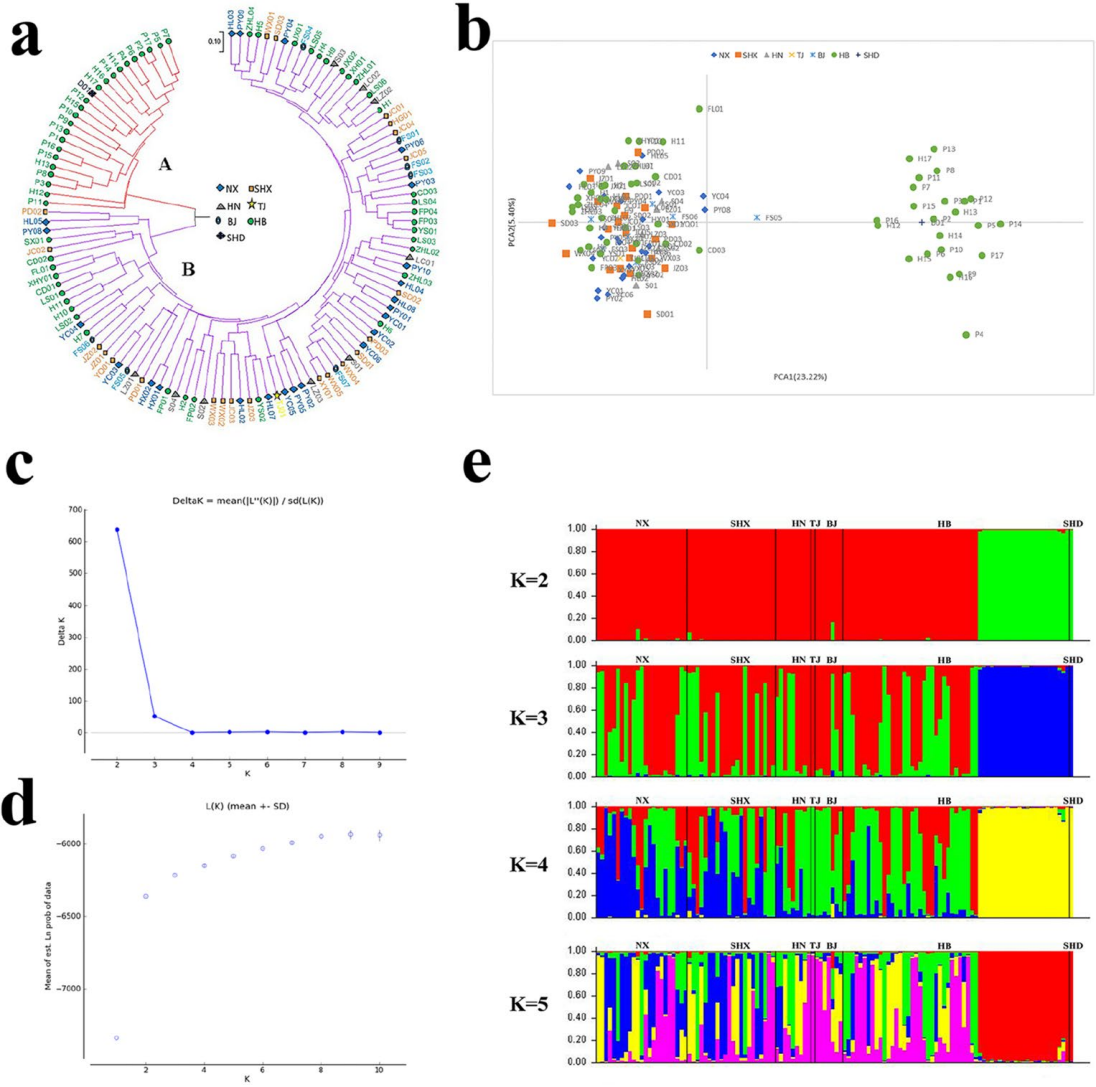
Structure analysis of 120 *A. altissima* var. *erythrocarpa* individuals. (**a**) Dendrogram based on the genetic distance of individuals in different populations (UPGMA); (**b**) principal coordinate analysis plot; (**c**) delta* K* distribution based on the rate of change in L(*K*) between consecutive* K* values. (**d**) Estimated average likelihood L(K) distribution(mean ± SD) from 2 to 10 possible clusters (K). (**e**) histogram of the structure assignment when *K* = 2–5.

According to the genetic similarity coefficients among the test materials of *A. altissima* var. *erythrocarpa*, using MEGA7 software, the UPGMA algorithm was used to constructed a dendrogram for 120 *A. altissima* var. *erythrocarpa* materials (Fig. 5a), which could be divided into two groups (A and B) through cluster analysis. Group A was mainly composed of 24 materials from the Handan and Pingshan areas of Hebei Province and Shandong Province, accounting for 20% of the total test materials. There were 96 materials from the 7 natural populations of *A. altissima* var. *erythrocarpa* in Group B, accounting for 80% of the total materials tested. In Group B, the test materials from Henan Province were scattered in each subgroup, while those in Hebei Province were more clustered, which may be related to the genetic background of the samples and the environment at the sample collection sites. Using GenAlEx 6.502 software to carry out principal coordinate analysis (Fig. 5b), the variation contribution rates of the first and second principal components were 23.22% and 5.4%, respectively, and the classification results were consistent with those of the cluster analysis. This further proves that the primers developed in this study are effective and will not mistakenly indicate obvious inter-population variation due to genetic drift. STRUCTURE 2.3.4 The software results were processed by Structure Harvester and found that (Fig. 4d) the value of LnP(*D*) continued to increase, there was no inflection point, and the Delta *K* values could be used to determine the optimal *K* value (Supplementary Table 3, Fig. 5c,d). STRUCTURE analysis showed that the seven natural populations of *A. altissima* var. *erythrocarpa* could be genetically divided into two different subpopulations using the *ΔK* method (Fig. 5c), and the clustering pattern was the same as that in the dendrogram. When* K* was equal to 3–5, some individuals in subgroup 1 were divided into several subgroups, indicating that these individuals were hybrids and that the other individuals had a pure pedigree (Fig. 5e). Individuals HX01, WX04, and ZHL04 in subgroup 1 consistently originated from two subgroups. The individuals in subgroup 2 originated from a single ancestral group, except that P15 and P16 were divided among three subgroups (*K* = 3–5).

## Discussion

In recent years, SSR molecular markers have been widely used in studies of species genetic diversity due to their advantages, such as high species specificity and cross-related species transfer, and primer development is their primary use. To date, only JosphatK. Saina et al.^18,33^ identified 219 SSR loci based on the chloroplast genome of *A. altissima* (Mill.) Swingle in 2018. In 2021, 13 EST-SSRs were developed, and 10 polymorphic chloroplast microsatellite (cpSSR) markers were constructed based on the transcriptome of *A. altissima* (Mill.) Swingle and 219 cpSSR markers. The distribution characteristics of cpSSRs and EST-SSRs were similar, but cpSSRs did not show hexanucleotide and compound nucleotide repetitions. In this study, 10,681 potential microsatellite loci were obtained based on transcriptome sequencing results of tissue-cultured seedlings from young leaves of *A. altissima* var. *erythrocarpa*, which was far lower than that reported by JosphatK. Saina et al.^18^ (33,084 potential SSR loci), and the EST-SSR distribution characteristics were similar. The SSR markers developed in this study had a high effective amplification rate, which is helpful for analyzing the genetic background and performing auxiliary breeding of *A. altissima* var. *erythrocarpa*.

Most plants show mononucleotides, dinucleotides and trinucleotides as dominant repeat motifs, such as *Stephanandra incisa*^23^, *Bougainvillea cultivars*^21^, *Cocos nucifera* L.^34^, *A. altissima* (Mill.) Swingle^18^, *Pinus bungeana*^11^, *Ulmus pumila* L.^35^, and *A. altissima* var. *erythrocarpa.* The results of the transcriptome analysis showed the same pattern, with these three types accounting for 90.12% of the total SSRs. AG/CT is the most frequent dinucleotide repeat motif in most plants, and the homopurine–homopyrimidine extension is often found in the 5′ untranslated region, which plays an important role in gene expression and nucleic acid metabolism regulation in plants. AAG/CTT is the main nucleotide repeat type in dicotyledons^18,23,24,33,36–41^. In this study, the dinucleotide AT/AT repeat type had the highest frequency, which was consistent with the results for *Pinus bungeana*^11,42^, *Bougainvillea* cultivars^21^. The majority of the trinucleotide repeats were AGA/TCT, followed by AAG/CTT. Among the 140 pairs of primers screened and synthesized, 106 yielded amplification bands, and the effective amplification rate reached 75.71%, which was similar to that in *A. altissima* (Mill.) Swingle (80%)^18^ and *Cinnamomum chago* (70.59%)^43^. The polymorphism rate of the primers was 13.57%, which was significantly lower than that reported by Saina et al.^18^ (65%), which may be caused by the use of more randomly selected primers, small genetic differences between samples and different environmental selection pressure. Therefore, EST-SSRs are still the most economical and effective tool for developing a large number of primers at the same time using transcriptome data. The chloroplast genome of *A. altissima* is a circular molecule with a size of 160,815 base pairs and a tetrad structure. The chloroplast genome size of *A. altissima* was similar to that of *Arabidopsis thaliana* (L.) Heynh. (120–217 kb) and *Populus trichocarpa* (155–159 kb), so it was relatively easy to develop cpSSR markers for *A. altissima*. Compared with cpSSRs, EST-SSRs exist in the gene regions of the nuclear genome and are highly polymorphic^18,44^. In this study, EST-SSRs were combined with molecular markers of *A. altissima* var. *erythrocarpa* to study the point mutations in *A. altissima* var. *erythrocarpa*, providing important resources for subsequent germplasm resource protection and evolutionary research in populations. Phenotypic traits combined with molecular markers could be used to analyze the correlations of traits, providing guidance for the utilization of germplasm resources and breeding of new varieties.

*PIC* can measure the polymorphism of primers: *PIC* > 0.5 indicates highly polymorphic, 0.25 < *PIC* < 0.5 indicates moderately polymorphic, and *PIC* < 0.25 indicates weakly polymorphic^45^. In this study, 19 pairs of clearly polymorphic primers with the expected size were screened, and the average *PIC* value was 0.671, lower than the average *PIC* value of *A. altissima* (0.819)^18^. *PIC* > 0.5 indicated that the identified SSR loci had high genetic resolution, making them conducive to population genetic analysis and revealing that the studied *A. altissima* var. *erythrocarpa* accessions had high genetic diversity. This may be related to the complex living environment of wild *A. altissima* var. *erythrocarpa*. The materials of wild *A. altissima* var. *erythrocarpa* used in this experiment are widely distributed in Ningxia, Shanxi, Shandong, Henan, Tianjin, Beijing, Hebei and other places. The sampling points were mostly on hillsides, on roadsides and in other similar places. The diversity of the geographical environment and the characteristics of climate variability jointly determine the diversity of the living environment of *A. altissima* var. *erythrocarpa*. Therefore, this study further reveals the genetic diversity and structure of this species.

The average expected heterozygosity is less affected by sampling than other metrics. The higher the value is, the lower the genetic consistency and the higher the level of genetic diversity. The average expected heterozygosity of *A. altissima* var. *erythrocarpa* from 7 natural populations was 0.509, similar to the results of Saina et al.^18^, which may be related to the wide distribution and complex living environment of wild *A. altissima* var. *erythrocarpa*. The mean *He* was slightly lower than that reported in previous studies^18,28,30^, which may be related to the fact that *A. altissima* var. *erythrocarpa* is a variety of *A. altissima* and originated in the same place. In the study of Saina et al.^18^, the mean *Ne* of *A. altissima* (Mill.) Swingle is 3.757. In this study, the mean *Ne* of *A. altissima* var. *erythrocarpa* is 2.838. This may be due to the fact that the population size (14) and sample size (165) of *A. altissima* (Mill.) Swingle are larger than that of this study (7 and 120), and the distribution range (99–120° N and 29–37° E) is wider than that of *A. altissima* var. *erythrocarpa* (105–117° N and 33–41° E). Among the seven natural populations, HB (*Ne* = 4.439, *I* = 1.625) had the highest genetic diversity, TJ (*Ne* = 1.421, *I* = 0.292) and SHD (*Ne* = 1.421, *I* = 0.292) had the lowest genetic diversity. This may be because each group has different site conditions (city streets, mountains, rocks), and its growing environment is different; Samples from city streets, there is human interference. These all influence the measurement of genetic diversity. The *F*_*ST*_ comparison between nineteen pairs of EST-SSRs and 7 natural populations (*Fst* mean = 0.163, *Nm* mean = 3.783) showed a high degree of genetic differentiation (Table 4 and Fig. 4) and frequent gene exchange, indicating that there was strong gene flow (Nm > 1) among the seven natural populations, which inhibited genetic differentiation caused by genetic drift, with low differentiation level and high intra-population differentiation level. This is supported by PCoA (Fig. 5b) and dendrogram based on the UPGMA algorithm (Fig. 5a).

According to genetic distance and genetic similarity analysis, NX and SHX had the closest genetic relationship. To effectively analyze *A. altissima* var. *erythrocarpa* germplasm resources, information about natural habitat, genetic evolution, genetic diversity, and the degree of species mixing is required^46^. For principal coordinate analysis, structure analysis, and cluster analysis based on genetic distance as determined by UPGMA, the seven natural populations of red-fruited *Ailanthus* could be divided into two subgroups; one was subgroup A dominated by materials from Handan and Pingshan County, Hebei Province, and the other was subgroup B (Fig. 5a,b,e) dominated by materials other than those from SHD, which may be because the material for transcriptome sequencing was taken from Handan, and subgroup A was highly represented among the samples. Structure-based clustering is a reliable tool for estimating the degree of species mixing, and the kinship of a small number of samples in subpopulation A and subpopulation B was complex (Fig. 5e), indicating that during the evolution of the species, frequent gene exchange and recombination between individuals in the region led to a richer genetic structure. Some germplasm resources are introduced to different places via wind and animal and human activities. NX, SHX, HN, TJ, and BJ were clustered in subgroup A, which may not be geographically isolated, and subgroup A and subgroup B included samples of the HB natural population. When *K* = 3–5, individuals HX01, WX04, and ZHL04 in subgroup 1 have complex lineages and are always related to both subpopulations. Individuals in subgroup 2, except for P15 and P16, which can be divided into three subgroups, all originated from a single ancestral group, these results can explain that there is no geographical separation between the 7 natural groups, and the kinship and complexity of each individual can be clearly known. The resources of the same area were almost always clustered together (Fig. 5a,b,e). This finding is similar to the classification results of *Astragali Radix*^47^, indicating high genetic similarity and frequent gene exchange.

DNA fingerprinting technology analyzes the genetic material itself, avoiding the influences of environmental factors, sample morphology, material source and other factors, and identifies the germplasm genotypes as indicators at the molecular level. It has been widely used in grain, oil, vegetable and fruit crops, flowers, shrubs, arbors and other plants^16,20,21,48^. DNA fingerprinting has high simplicity and stability, variability, and multiple loci and can distinguish members of the same family, such as different species, varieties, and forms and even the same strain and subtle variation between individuals. It can be used in germplasm genetic relationship inference, genetic breeding, population genetic structure determination, ecological and evolutionary studies, classification and other valuable genetic marker methods^48–50^. However, there have been no reports on the fingerprint information of *A. altissima* var. *erythrocarpa*. In this study, four of nineteen EST-SSR core primers (p33, p15, p46 and p92) were screened to distinguish 120 tested *A. altissima* var. *erythrocarpa* materials and construct a unique molecular ID card. The establishment of fingerprinting for *A. altissima* var. *erythrocarpa* is of great significance for its subsequent breeding, as the resource can be used to identify varieties and excellent genes as well as their variation and transmission in offspring, and lays a foundation for the establishment and improvement of fingerprinting databases of *A. altissima* var. *erythrocarpa* variety resources, genetic resource management and intellectual property protection. However, in this study, there were few test materials, the sample collection area was only in northern China, and the fingerprint background of the "core germplasms” of *A. altissima* var. *erythrocarpa* lacked in-depth research. As a result, further verification of the reliability of the results is required.

## Conclusion

In this study, nineteen pairs of EST-SSRs revealed high genetic diversity and significant genetic structure in *A. altissima* var. *erythrocarpa*. Using four pairs of SSR core primers, a total of 120 wild individuals from 7 regions of *A. altissima* var. *erythrocarpa* were 100% distinguished and assigned unique identification IDs. The experimental resources of *A. altissima* var. *erythrocarpa* have rich genetic variations, mainly derived from intraspecific variations, and do not exhibit geographical variation. The research results presented in this article provide a public resource of SSR primers in *A. altissima* var. *erythrocarpa*, in turn providing a theoretical basis for effective management, protection, and utilization of its germplasms. The excellent varieties studied according to the SSR primers of *A. altissima* var. *erythrocarpa* can be introduced by biotechnological means and can also be preserved ex situ or in situ, which would be conducive to subsequent research. The findings also provide genetic resources for an in-depth understanding of population genetics and evolutionary history, joint genomics research, the selection of dominant parents for the cultivation of new varieties, and intellectual property protection in *A. altissima* var. *erythrocarpa*.

## Materials and methods

### Plant materials

**Figure 6:**
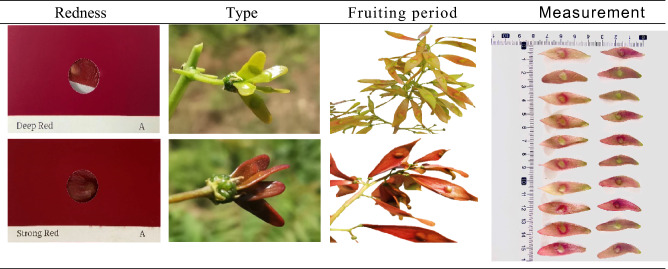
Screening index system for 120 *A. altissima* var. *erythrocarpa* individuals. Redness: degree of redness of the samara; type: full red and gradually red; fruiting period: early red (June-July) and late red (July August); measurement: large fruit and small fruit.

**Figure 7:**
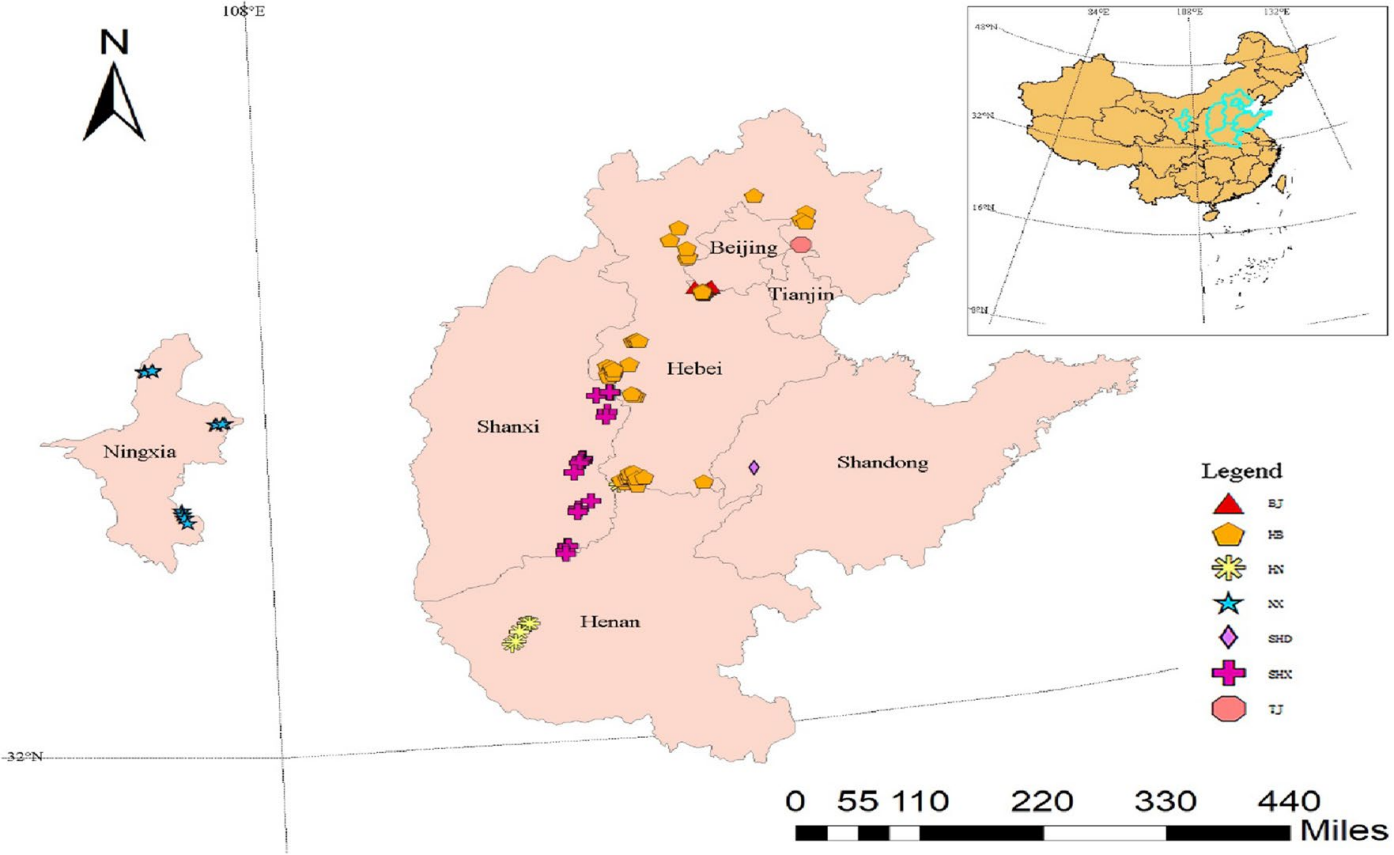
Geographic distribution of 120 *A. altissima* var. *erythrocarpa* wild individual samples from 7 provinces in China. NX: Ningxia Province, China; SHX: Shanxi Province, China; HN: Henan Province, China; TJ: Tianjin Province, China; BJ: Beijing Province, China; HB: Hebei Province, China; and SHD: Shandong Province, China. The map was generated using ArcMap 10.2 software.

A total of 120 wild individuals of *Ailanthus altissima* var.* erythrocarpa* selected based on four indicators were collected from 7 provinces in China (Ningxia, Shanxi, Henan, Tianjin, Beijing, Hebei, and Shandong). The geographical locations of the sample spanned 106.00–117.72 east longitude, 33.76–41.12 north latitude, and 34.7–1858.2 m altitude, and the local soil was mostly red soil. When sampling, the latitude, longitude and altitude of the sample were recorded with a GPS device. The HN, TJ, BJ and SHD sampling sites were distributed discontinuously, with 6–10 plants per hectare. The number of individuals was small., the distribution was scattered, and the distance between the trees collected was not less than 50 m to prevent half-sib relationship between the samples. (Supplementary Table 1, Figs. 6 and 7).

### DNA extraction

Fresh and tender *Ailanthus altissima* var. *erythrocarpa* leaves were selected and brought back into the laboratory in an ice box. Genomic DNA of *Ailanthus altissima* var. *erythrocarpa* was extracted by the improved CTAB method and an Omega kit. DNA concentration and quality were determined by a microspectrophotometer (model: Nano300). After quality determination, the DNA was diluted to 30 ng/μl in each tube. A total of 5 tubes (150 μl/tube) were used as working liquid. The original liquid and working liquid were stored in a freezer at − 20 °C for later use.

### Development of SSR primers

Due to the lack of a reference genome sequence, leaves of tissue-cultured plantlets were sent to Wuhan Matwell Biotechnology Co., Ltd., for transcriptome sequencing and primer design. SSR primer design data were provided by Professor Dukejiu of Hebei Agricultural University. A total of 140 pairs of primers were randomly selected from 10,681 pairs of primers by Excel, Sequon Bioengineering (Shanghai) Co., Ltd., and Tsingke Biotechnology Co., Ltd., were commissioned to synthesize primers.

### SSR primer screening and PCR amplification

First, the mixtures of 8 samples were used as templates to screen out primers with clear and bright bands that matched the size of the product. Second, primers with clear bands and good polymorphism were selected for amplification in 6 samples. Finally, the core primers were screened from 120 samples, which were verified with the primers.

1. Nondenaturing polyacrylamide gel electrophoresis. The optimized PCR system was carried out in a volume of 20 µl: DNA template 1.5 µl (30 ng/μl), 2 × Taq PCR Master Mix 10 μl, upstream and downstream primers 1 μl (10 µmol/µl), and ddH_2_O 6.5 µl. The thermal cycling parameters used in the PCR procedure were 95 °C predenaturation for 5 min, followed by 33 cycles of 95 °C for 1 min, 55 °C annealing for 30 s, and 72 °C extension for 45 s, with a final elongation step at 72 °C for 8 min. The samples were then stored at 4 °C. The initial screening conditions for polymorphic primers were as follows: 2% agarose gel electrophoresis, voltage 110 V, 30 min, real-time photography; screening and verification with 8% nondenatured polyacrylamide gel electrophoresis, 200 V, 100 mA, 20 W, 1 h and 300 V, 100 mA, 20 W, 2.5 h; silver staining (AgNO_3_); and photo storage for subsequent analysis.
2. Fluorescent capillary electrophoresis The PCR systems were similar to those used for nondenaturing polyacrylamide gel electrophoresis, with the only modification being the addition of 1 μl (10 µmol/μl) fluorescent primer (FAM/TAMRA/HEX/POX) to each plate system. The procedure was as follows: 95 °C predenaturation (5 min), followed by 25 cycles of 95 °C denaturation (1 min), 58 °C annealing (30 s), and 72 °C extension (45 s) and then 10 cycles of 95 °C denaturation (1 min), 58 °C annealing (30 s), and 72 °C extension (45 s), with a final elongation step at 72 °C for 8 min. The products amplified by PCR were sent to Taihe Biotechnology Co., Ltd., for testing.

### Data analysis

The fluorescence capillary electrophoresis detection results were viewed using GeneMarker V2.4.0 software and collated in Excel. GenAlEx 6.502 software was used to calculate the average number of alleles (*Na*), average effective number of alleles (*Ne*), Shannon's information index (*I*), average observed heterozygosity (*Ho*), average expected heterozygosity (*He*), and fixation index (*F*). Hardy–Weinberg equilibrium tests were performed^18,51^. The polymorphism information content (*PIC*) was calculated using Cervus 307 software^18,51^. In GenAlExv6.5, *F* and *Nm* (*Nm* = (0.156/^S^H_UA_)^2^ statistical calculations (*Fis*, *Fit* and *Fst*) and principal coordinate analysis (PCoA) were performed in combination with Microsoft Excel. The paired heat map of Fst and Nm was constructed in R 4.2.2. Used Populations v1.2.32 to calculate genetic distances, based on genetic similarity coefficients, a phylogenetic tree was drawn using MEGA7^52^. Mixed analysis based on Bayesian models was used for population structure analysis in Structure v2.3.4^53^. Then, the number of Markov chain Monte Carlo (MCMC) iterations after burn-in was set to 100,000, with a run length of 100,000 and a genetic uniform cluster number (*K* value) between 1 and 10. Each analysis consisted of 10 repeated runs. The optimal *K* value was determined using the best-*K* method in StructureHarvester^54^. Then used CLUMPP Windows.1.1.2b software^55^ to found the real *K* value, replaced the *K* value in STRUCTURE, and generated a genetic structure map.

### Statement

Permission to collect plant samples was obtained for this study. Voucher specimens were identified by Alfred Rehder and deposited in the Chinese Field Herbarium (CFH). This study complied with local and national guidelines.

### Supplementary Information

Supplementary Information.

## Data Availability

The dataset is available from the NCBI BioProject (PRJNA1006443) and NCBI Short Read Archive (SRA) with accession number SRR25900069.
