# Supplementary material for: Genetic diversity, population structure, and DNA fingerprinting of Ailanthus altissima var. erythrocarpa based on EST-SSR markers
Source: Sci Rep. 2023 Nov 7;13:19315. doi: 10.1038/s41598-023-46798-2 (PMC10630516; doi:10.1038/s41598-023-46798-2)
Supplement: Supplementary file 1 — Supplementary Information. [file 41598_2023_46798_MOESM1_ESM.docx]

**Genetic diversity, population structure, and DNA fingerprinting of *Ailanthus altissima* var. *erythrocarpa* based on EST-SSR markers**

**Manman Zhang ^1, 3^, Conghui Zheng ^2, 3^, Jida Li^1^, Xueyong Wang ^2, 3^, Chunpeng Liu ^2, 3^, Xiangjun Li^2, 3^, Zhenhua Xu^2, 3^ ^*^ & Kejiu Du^1 *^**

^1^Hebei Agricultural University, Baoding 071000, Hebei, China.

^2^Hebei Academy of Forestry and Grassland Sciences, Shijiazhuang 050061, Hebei, China.

^3^Hebei Technical Innovation Center for Forest Improved Variety, Shijiazhuang 050061, Hebei, China. * E-mail: hblky303@126.com.

**Supplementary table 1 Geographic information of 120 wild individual samples of Ailanthus rubrum**

| Sample | Pop | lng | lat | Hight(m) |
| --- | --- | --- | --- | --- |
| HX01 | NX | 106.563766 | 36.287642 | 1829.4 |
| HX02 | NX | 106.563766 | 36.287642 | 1830.2 |
| HL02 | NX | 105.989348 | 38.713237 | 1433.3 |
| HL03 | NX | 105.996257 | 38.704474 | 1378 |
| HL04 | NX | 106.119484 | 38.723639 | 1136.9 |
| HL05 | NX | 106.121285 | 38.727045 | 1135.4 |
| HL07 | NX | 106.120852 | 38.726681 | 1135.8 |
| HL08 | NX | 106.129074 | 38.739437 | 1134.5 |
| YC01 | NX | 107.319725 | 37.787959 | 1388.5 |
| YC02 | NX | 107.159571 | 37.775953 | 1544.2 |
| YC03 | NX | 107.159831 | 37.775988 | 1544.9 |
| YC04 | NX | 107.184965 | 37.775312 | 1580.7 |
| YC05 | NX | 107.286266 | 37.785364 | 1426.5 |
| YC06 | NX | 107.287921 | 37.785375 | 1436.1 |
| PY01 | NX | 106.571414 | 36.236632 | 1798.0 |
| PY02 | NX | 106.571193 | 36.237060 | 1793.7 |
| PY03 | NX | 106.570329 | 36.226143 | 1803.8 |
| PY04 | NX | 106.597822 | 36.180087 | 1824.6 |
| PY05 | NX | 106.597822 | 36.180087 | 1824.8 |
| PY06 | NX | 106.607675 | 36.172259 | 1791.2 |
| PY08 | NX | 106.652356 | 36.073872 | 1858.2 |
| PY09 | NX | 106.652325 | 36.074344 | 1854.4 |
| PY10 | NX | 106.652031 | 36.071571 | 1843.7 |
| YQ01 | SHX | 113.581849 | 37.924679 | 814.7 |
| PD01 | SHX | 113.837264 | 37.958076 | 399.1 |
| PD02 | SHX | 113.831389 | 37.960375 | 415.9 |
| PD03 | SHX | 113.811211 | 37.963081 | 462.7 |
| JZ01 | SHX | 113.721904 | 37.632900 | 897.4 |
| JZ02 | SHX | 113.726059 | 37.627761 | 887.5 |
| JZ03 | SHX | 113.685554 | 37.548787 | 948.7 |
| WX01 | SHX | 113.210410 | 36.862529 | 1109.3 |
| WX02 | SHX | 113.205570 | 36.856651 | 1109.1 |
| WX03 | SHX | 113.194853 | 36.822440 | 1059.3 |
| WX04 | SHX | 113.172776 | 36.817840 | 1053.5 |
| WX05 | SHX | 113.145279 | 36.798123 | 1003.8 |
| XY01 | SHX | 113.037893 | 36.633633 | 978.0 |
| SD01 | SHX | 113.034601 | 36.005471 | 1000.9 |
| SD02 | SHX | 113.012888 | 35.973684 | 1002.2 |
| SD03 | SHX | 113.013730 | 35.948369 | 1035.3 |
| JC01 | SHX | 112.777264 | 35.378110 | 849.7 |
| JC02 | SHX | 112.784949 | 35.370311 | 849.0 |
| JC03 | SHX | 112.784780 | 35.370571 | 855.4 |
| JC04 | SHX | 112.729189 | 35.291958 | 969.5 |
| JC05 | SHX | 112.736348 | 35.245612 | 720.9 |
| HG01 | SHX | 113.254416 | 36.127999 | 1072.2 |
| S01 | HN | 111.984915 | 34.098366 | 669.2 |
| S02 | HN | 111.986093 | 34.097594 | 660.7 |
| S03 | HN | 112.031072 | 34.096963 | 578.1 |
| S04 | HN | 111.851911 | 33.956795 | 525.3 |
| LC01 | HN | 111.715000 | 33.759166 | 689.73 |
| LC02 | HN | 111.774066 | 33.802413 | 779.8 |
| LZ01 | HN | 113.750056 | 36.363623 | 404.2 |
| LZ02 | HN | 113.748708 | 36.363620 | 428.6 |
| LZ03 | HN | 113.749036 | 36.364135 | 415.1 |
| TJ01 | TJ | 117.508609 | 40.174892 | 213.1 |
| FS01 | BJ | 115.760768 | 39.595175 | 157 |
| FS02 | BJ | 115.812184 | 39.623989 | 275.9 |
| FS03 | BJ | 115.813639 | 39.62101 | 303.2 |
| FS04 | BJ | 115.824812 | 39.624389 | 333.23 |
| FS05 | BJ | 115.820943 | 39.635545 | 194.9 |
| FS06 | BJ | 115.697209 | 39.579498 | 120.91 |
| FS07 | BJ | 115.543686 | 39.648597 | 172.9 |
| SX01 | HB | 113.859049 | 36.379983 | 327.8 |
| FP01 | HB | 114.34104 | 38.818759 | 220.5 |
| FP02 | HB | 114.375988 | 38.823163 | 226.8 |
| FP03 | HB | 114.399633 | 38.835843 | 305.9 |
| FP04 | HB | 114.436676 | 38.814152 | 307.1 |
| YS01 | HB | 114.249343 | 37.866731 | 277.6 |
| YS02 | HB | 114.249629 | 37.867312 | 272.0 |
| JX01 | HB | 114.205313 | 37.875210 | 475.7 |
| JX02 | HB | 114.179870 | 37.901951 | 375.2 |
| LS01 | HB | 115.675186 | 39.520775 | 110.7 |
| LS02 | HB | 115.675659 | 39.522035 | 109.3 |
| LS03 | HB | 115.669239 | 39.524646 | 130.4 |
| LS04 | HB | 115.654678 | 39.526264 | 222.4 |
| LS05 | HB | 115.661504 | 39.569672 | 118.18 |
| LS06 | HB | 115.653509 | 39.550775 | 134.5 |
| ZHL01 | HB | 115.498262 | 40.157800 | 917.4 |
| ZHL02 | HB | 115.520104 | 40.186794 | 755.7 |
| ZHL03 | HB | 115.525913 | 40.191739 | 746.3 |
| ZHL04 | HB | 115.529114 | 40.328414 | 516.2 |
| XH01 | HB | 115.450083 | 40.704373 | 1020.0 |
| XHY01 | HB | 115.25395 | 40.506201 | 563.4 |
| FL01 | HB | 116.86135 | 41.121433 | 505.0 |
| CD01 | HB | 117.721354 | 40.721488 | 504.3 |
| CD02 | HB | 117.607428 | 40.612995 | 691.3 |
| CD03 | HB | 117.660825 | 40.552892 | 473.3 |
| H1 | HB | 113.798784 | 36.442328 | 529.5 |
| H2 | HB | 113.893376 | 36.458912 | 462.7 |
| H4 | HB | 113.931512 | 36.475364 | 512.9 |
| H5 | HB | 113.93204 | 36.489288 | 532.1 |
| H6 | HB | 113.931858 | 36.490739 | 537.0 |
| H7 | HB | 113.931858 | 36.490739 | 530.43 |
| H9 | HB | 113.95952 | 36.542148 | 616.4 |
| H10 | HB | 113.95952 | 36.542148 | 623.2 |
| H11 | HB | 114.017731 | 36.548147 | 378.5 |
| H12 | HB | 114.0314 | 36.372236 | 328.3 |
| H13 | HB | 114.0314 | 36.372236 | 330.4 |
| H14 | HB | 114.041632 | 36.331576 | 378.7 |
| H15 | HB | 114.047888 | 36.448132 | 382.2 |
| H16 | HB | 114.17212 | 36.450944 | 184.1 |
| H17 | HB | 115.131006 | 36.285150 | 45.3 |
| P1 | HB | 113.833267 | 38.283788 | 347.7 |
| P2 | HB | 113.839989 | 38.418625 | 450.0 |
| P3 | HB | 113.841456 | 38.418472 | 407.1 |
| P4 | HB | 113.837860 | 38.418552 | 395.0 |
| P5 | HB | 113.837858 | 38.418504 | 393.6 |
| P6 | HB | 113.837858 | 38.418503 | 387.1 |
| P7 | HB | 113.852751 | 38.241012 | 369.7 |
| P8 | HB | 113.892229 | 38.347192 | 229.5 |
| P9 | HB | 114.21308 | 38.42178 | 192.8 |
| P10 | HB | 113.900104 | 38.356357 | 233.9 |
| P11 | HB | 113.83144 | 38.283544 | 290.4 |
| P12 | HB | 113.930184 | 38.280564 | 194.6 |
| P13 | HB | 113.832368 | 38.238848 | 198.6 |
| P14 | HB | 113.91828 | 38.273612 | 219.2 |
| P15 | HB | 113.933267 | 38.352761 | 220.5 |
| P16 | HB | 113.915224 | 38.388332 | 306.5 |
| P17 | HB | 113.943264 | 38.347494 | 207.4 |
| D01 | SHD | 115.989994 | 36.434617 | 34.7 |

Supplementary table 2 ***Fst* and *Nm* values detected for population pairs of *A. altissima* var. *erythrocarpa.***

| Comparison | *F_ST_* | *Nm* |
| --- | --- | --- |
| NX VS. SHX | 0.016 | 15.401 |
| NX VS. HN | 0.030 | 7.978 |
| NX VS. TJ | 0.194 | 1.037 |
| NX VS. BJ | 0.053 | 4.441 |
| NX VS. HB | 0.040 | 6.043 |
| NX VS. SHD | 0.297 | 0.592 |
| SHX VS. HN | 0.025 | 9.907 |
| SHX VS. TJ | 0.199 | 1.008 |
| SHX VS. BJ | 0.044 | 5.432 |
| SHX VS. HB | 0.038 | 6.335 |
| SHX VS.SHD | 0.305 | 0.570 |
| HN VS. TJ | 0.200 | 0.998 |
| HN VS. BJ | 0.049 | 4.886 |
| HN VS. HB | 0.040 | 6.043 |
| HN VS. SHD | 0.304 | 0.573 |
| TJ VS. BJ | 0.183 | 1.115 |
| TJ VS. HB | 0.197 | 1.019 |
| TJ VS. SHD | 0.600 | 0.167 |
| BJ VS. HB | 0.051 | 4.636 |
| BJ VS. SHD | 0.342 | 0.480 |
| HB VS. SHD | 0.226 | 0.854 |
| Mean | 0.163 | 3.783 |

Supplementary table 3 Evanno table based on the STRUCTURE result file

| K | Reps | Mean LnP(K) | Stdev LnP(K) | Ln’(k) | ׀Ln’’(K)׀ | Delta K |
| --- | --- | --- | --- | --- | --- | --- |
| 1 | 10 | -7338.900000 | 0.623610 | — | — | — |
| 2 | 10 | -6362.140000 | 1.296320 | 976.760000 | 827.660000 | 638.469105 |
| 3 | 10 | -6312.040000 | 1.641273 | 149.100000 | 84.470000 | 51.466142 |
| 4 | 10 | -6148.410000 | 11.156906 | 64.630000 | 2.660000 | 0.238417 |
| 5 | 10 | -6081.120000 | 10.095191 | 67.290000 | 15.860000 | 1.571045 |
| 6 | 10 | -6029.690000 | 5.656746 | 51.430000 | 13.540000 | 2.393602 |
| 7 | 10 | -5991.800000 | 9.003333 | 37.890000 | 5.460000 | 0.606442 |
| 8 | 10 | -5948.450000 | 12.241936 | 43.350000 | 28.800000 | 2.352569 |
| 9 | 10 | -5933.900000 | 32.317522 | 14.550000 | 19.780000 | 0.612052 |
| 10 | 10 | -5939.130000 | 36.938238 | -5.230000 | — | — |

**Supplementary Figure 1** Fingerprints of 120 individuals of *A. altissima* var. *erythrocarpa*. A: p33; B: p15; C: p46; C: p92.

**
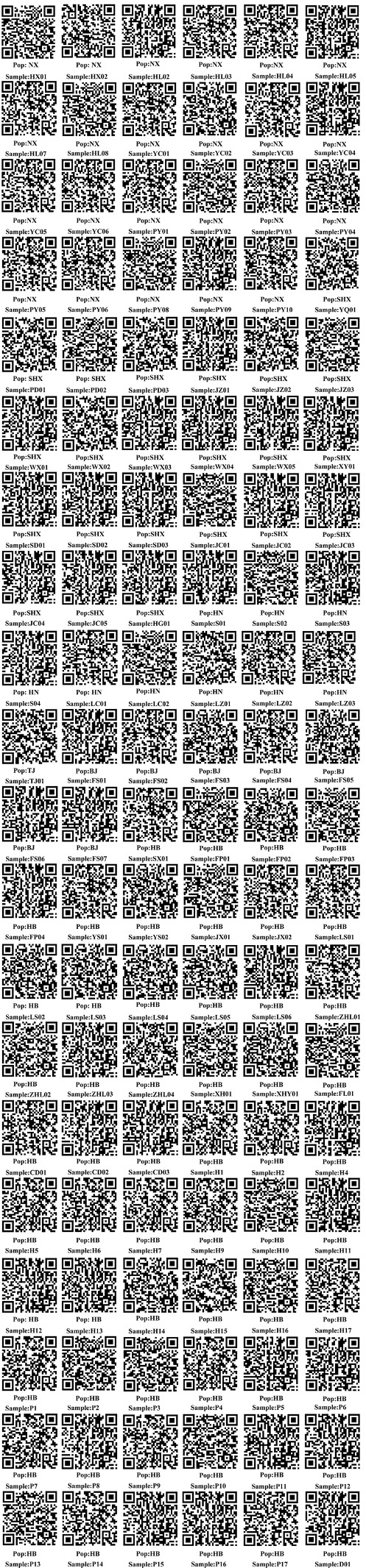
**
